# Perioperative Propranolol Against Dental Anxiety: A Randomized Controlled Trial

**DOI:** 10.3389/fpsyt.2022.842353

**Published:** 2022-02-21

**Authors:** Serge A. Steenen, Naichuan Su, Roos van Westrhenen, Arjen J. van Wijk, Daniël S. L. Tjia, Jan de Lange, Ad de Jongh

**Affiliations:** ^1^Department of Oral and Maxillofacial Surgery, Amsterdam University Medical Centers/Academic Center for Dentistry (ACTA), University of Amsterdam, Amsterdam, Netherlands; ^2^Department of Oral Public Health, Academic Centre for Dentistry Amsterdam, University of Amsterdam and VU University Amsterdam, Amsterdam, Netherlands; ^3^Department of Psychiatry, Parnassia Psychiatric Institute, Amsterdam, Netherlands; ^4^Department of Psychiatry and Neuropsychology, Maastricht University Medical Center, Maastricht, Netherlands; ^5^Institute of Psychiatry, Psychology & Neuroscience (IoPPN), King's College London, London, United Kingdom; ^6^Institute of Health and Society, University of Worcester, Worcester, United Kingdom; ^7^School of Psychology, Queen's University, Belfast, Ireland

**Keywords:** dental anxiety, phobic disorders, propranolol, randomized controlled trial, reconsolidation, extinction

## Abstract

**Background:**

Promising results from a trauma reactivation study on post-traumatic stress disorder suggest that propranolol is capable of attenuating symptoms of traumatically induced mental disorders by blocking memory reconsolidation.

**Methods:**

A randomized, parallel, placebo-controlled, quadruple-blind trial was designed to determine the effectiveness of perioperative propranolol during exposure to dental extractions in reducing dental anxiety in patients with dental anxiety or dental phobia. Between November 2014 and December 2018, 52 patients with high levels of fear in anticipation of dental extractions who were referred to a department of oral and maxillofacial surgery for at least two tooth and/or molar removals with 1 month in between were included. On the first visit participants received either 120 mg of perioperative oral propranolol (*n* = 19) or placebo (*n* = 17), and a core fear memory was reactivated 1 h preoperatively. The primary outcome was change in severity of dental anxiety from baseline to 1-month follow-up, as indexed by the short version of the dental anxiety inventory (S-DAI). Secondary outcome measures were change in intra-operative state anxiety and specific phobia diagnoses.

**Results:**

Linear mixed model (LMM) yielded no statistically significant difference in change of dental trait anxiety from baseline to 1-month follow-up between propranolol and placebo groups (Cohen's *d* = 0.23). S-DAI scores decreased in both study arms from baseline to follow-up (propranolol arm: from 32.1 [SD = 7.3] to 29.1 [SD = 8.8]; placebo arm: from 31.6 [SD = 7.5] to 27.1 [SD = 6.5]). Also, administering propranolol was not associated with a significant difference in change of intra-operative state anxiety or phobia diagnoses between groups over time.

**Conclusions:**

The results do not concur with earlier findings regarding post-traumatic stress disorder, and suggest that individuals with traumatically induced fears or phobias do not benefit from the application of perioperative propranolol.

## Introduction

Propranolol recently regained interest in the treatment of anxiety disorders when its effects on human fear memory storage were found (1–3). This indicated that after reactivation, previously consolidated fear memories can return to a transient labile state and as such become prone to pharmacological disruption; a ß-adrenergic and synaptic protein-dependent process in the basolateral amygdala called “memory reconsolidation” (4). Most compelling evidence for propranolol's effects on human fear memory reconsolidation originates from laboratory trials by Kindt et al. (3–8). They showed that, dependent on minor environmental changes that are difficult to control in clinical practice, fear memory reactivation may initiate three transitory phases: the first being labilization and reconsolidation; the second being stability; and the third being the generation of a new memory trace and extinction (6, 9–11). In 2018, Brunet et al. (12) showed propranolol to block fear memory reconsolidation in a clinical population. In this randomized placebo-controlled trial 60 chronic post-traumatic stress disorder (PTSD) patients received six weekly sessions of imaginal trauma reactivation, accompanied by oral propranolol administered 90 mins prior to reactivation (12). The results showed a substantial reduction in PTSD symptom severity when compared to placebo at 6 week (12), suggesting that progress could be made in those suffering from anxiety disorders, including specific phobias, that likewise develop from memories of negative past events.

One of the most common traumatically induced fears and phobias are the ones related to dental treatment with prevalence rates in Western countries of 24 and 4%, respectively (13). Because dentally anxious patients' behavior increases operative time and complicates postoperative recovery (14), the American Oral and Maxillofacial Surgery (OMFS) guideline (15) recommends to employ high-risk symptom relief with conscious or deep sedation. However, dental fear conditions are clearly rooted in the experience of previous negative (mainly dental) events (16–19) and disruption of crucial fear memories that underlie dental anxiety and dental phobia is likely to serve as an effective curative treatment. This may particularly hold true in patients with fear of extractions because these are among the most strongly feared and frequently employed procedures in dentistry (20). To this end, testing the effect of propranolol on individuals suffering from dental fear in anticipation to the actually feared situation (i.e., dental extractions) may provide an excellent example of a context for research into the applicability of this drug to other fears and phobias.

To assess the memory reconsolidation disrupting properties of propranolol in patients suffering from dental fear, we conducted a quadruple-blind, randomized, placebo-controlled clinical trial evaluating the effectiveness of propranolol in the reduction dental (trait) anxiety among patients with dental anxiety or dental phobia. We hypothesized that fear memory reactivation and administration of the active substance (i.e., two 40 mg propranolol capsules 1 h prior to dental extraction, followed by one 40 mg capsule immediately postoperatively) would result in a significantly greater reduction of dental anxiety (both state and trait anxiety) in patients with dental anxiety or dental phobia, compared to the effects of the placebo comparator, from baseline to 1-month follow-up appointment. Secondly, we predicted that the use of propranolol would result in a significantly greater decrease of specific (i.e., dental) phobia diagnoses compared to the placebo comparator, from baseline to the 1-month follow-up appointment.

## Materials and Methods

This trial was designed as a randomized, placebo-controlled, two-group, parallel, quadruple (participant, care provider, investigator and outcomes assessor) blind superiority trial of 36 participants with an allocation ratio of 1:1. This trial (protocol number NL42210.018.13) was conducted in accordance with the Declaration of Helsinki and received formal ethical approval by the Institutional Review Board of the Academic Medical Center on July 24, 2014 (application 2013_343). The trial is registered on ClinicalTrials.gov (NCT02268357) and on The Netherlands National Trial Register (NTR5364). All participants provided written informed consent at least 24 h after receiving full explanation of the procedures and were randomly allocated to receive oral propranolol or placebo. Full details of the trial protocol have been published elsewhere (14).

### Study Group

Potential participants were recruited *via* referrals to the department of OMFS of the Amsterdam University Medical Center—location Academic Medical Center, Amsterdam, the Netherlands.

To take part in the study, potential participants were required to have an indication for the removal of at least two teeth and/or molars which required two separate (i.e., baseline and 1-month follow-up) dental extraction appointments. Furthermore, potential participants were asked to rate how anxiety provoking a tooth or molar removal was to them on a four-point scale, ranging from 1 (“not anxiety provoking at all“); 2 (“more or less anxiety provoking“); 3 (“highly anxiety provoking“); to 4 (“extremely anxiety provoking”). Potential participants were eligible for inclusion if they indicated that a score of 3 or 4 applied to them. Potential participants were excluded if they: (I) had contra-indications for using propranolol (14), (II) used another β-adrenoreceptor antagonist; (III) used another anxiolytic or antidepressant medication; (IV) if they were currently in therapy for dental anxiety; or (V) had a systolic blood pressure of <100 mmHg.

### Study Procedures

**Figure 1:**
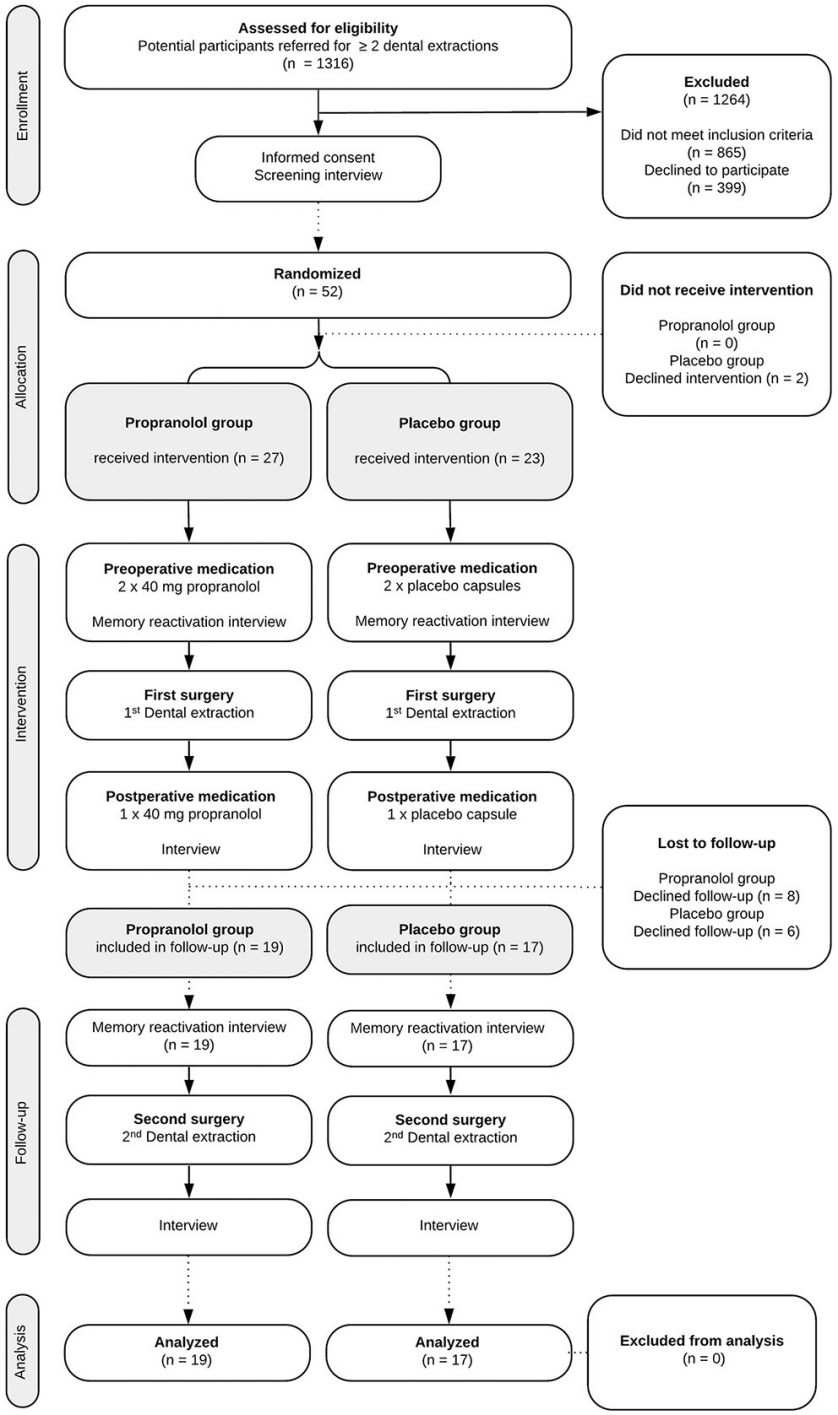
Flow-diagram of study protocol.

The study procedures and participant flow through each stage are summarized in Figure 1. To complete the study protocol, a participant was required to complete three stages (i.e., three separate hospital appointments). It is common practice not to anesthetize the mandible (inferior alveolar nerve) bilaterally; hence dental extractions such as third molar removals are often performed in two sessions, in this study with ~1 month in between.

Approximately 1 h prior to the first dental extraction, participants received two 40 mg oral propranolol capsules or visually identical placebo capsules directly before fear memory reactivation. Next, participants were interviewed and asked to recall their memory of a distressing event that initiated or exacerbated their dental anxiety in order to produce a grammatical first-person, chronological, script-driven mental imagery of the event. The interview focused on depicting the most aversive aspects of the narrative. Reconsolidation is expected to initiate 3–10 mins following the memory reactivation and to last for about 2–6 h (21). It has been previously shown that the higher the trait anxiety of an individual, the less likely it is that propranolol reduces fear by disrupting memory consolidation, e.g., Soeter and Kindt (22). Therefore, a relatively high total dose of perioperative propranolol was used in this study, with peak plasma concentration level expected at the time of surgery. Approximately 1 h after having recalled the fear memory, the participant was guided to the operating theater. We expected further reactivation of unintentional fear memory during the subsequent surgery, with maximum anxiety level at injection of the local anesthesia (23). Directly post-operatively, participants received the last capsule containing 40 mg of propranolol or placebo.

Shortly prior to the second dental extraction 1 month later, no study medication was administered. Approximately 30 mins after having recalled the memory of a distressing event that initiated or exacerbated their dental anxiety, the second surgery was performed.

### Randomization and Blinding

The random allocation sequence was computer-generated by the manufacturer of the investigational medicinal products (Good Manufacturing Practices-certified hospital pharmacy). Trial participants, investigators, data collectors, the statistician, and outcome assessors were blinded to the study-group assignments.

### Measures

#### Primary Effectiveness End Point

Severity of dental (trait) anxiety was indexed with the Dutch version of the Short version of the Dental Anxiety Inventory (S-DAI) (24).

#### Secondary Effectiveness End Points

##### Intra-operative State Anxiety

Patients were asked to rate the question “How much anxiety did you experience during the last treatment” using a standardized continuous self-report visual analog scale (VAS) ranging from “no anxiety at all” 0 to “extreme anxiety” 100.

##### Specific Phobia Diagnoses

The presence of specific (i.e., dental) phobia in accordance with the DSM-IV, TR criteria) were assessed using the Phobia Checklist, developed for the assessment of dental phobia (13). At present, dental phobia is still considered part of the blood-injection-injury (B-I-I) phobia subtype of specific phobia, although individuals with positive screen of dental phobia rate typical B-I-I-related stimuli as relatively little anxiety provoking (25). When the trial commenced this questionnaire was not yet updated to the DSM-5 criteria.

#### Tertiary Effectiveness End Points

Preoperatively, heart frequency and mean arterial pressure were recorded to assess the physiological ß-blocking effect of propranolol.

The Impact of Events Scale Revised (IES-R) (26), one of the most widely used self-report instruments for trauma-related symptoms, was used to assess symptoms of post-traumatic stress in the week before the dental extraction sessions. A score of 26 is considered the cut-off point for a clinically significant level of trauma-related symptomatology (27).

### Sample Size Calculation

A power analysis was performed for the primary outcome, the S-DAI. Power analysis is not possible for more complex analyses such as Linear Mixed Model (LMM) based on the available standardized software such as G^*^Power. The most comparable statistical method for which power analysis is available in this study, is the *F*-test (two-way ANOVA), which is therefore considered as the alternative. In order to detect a difference between the propranolol and control condition on changes in dental anxiety scores over time, a two-way (one-within and one-between subjects factor) repeated measures ANOVA was used. Using G^*^Power 3.0 software, assuming a correlation of 0.50 between two repeated measurements, a medium size treatment effect (f = 0.25), a power of 0.80, an α significance level of 0.05, and two treatment conditions, the power analysis results in a total sample size of 34 persons [17 persons per group; (14)].

#### Statistical Analysis Plan

The distributions of demographic variables and quantitative data are displayed in tables. The analysis was planned per protocol. Associations between categorical variables were analyzed using the χ^2^-test (two-tailed) for unpaired data, the Fisher's Exact test (two-tailed) for binary unpaired data if <80% of the cells in the contingency tables had an expected frequency of >5, and the McNemar's test (two-tailed) for paired binary data. The assumption of normality was met for all variables. Differences between the propranolol and placebo condition on continuous variables were analyzed using the independent-samples *t*-test (two-tailed) and differences between two consecutive measurements in time within one allocation group were analyzed using the paired *t*-test (two-tailed). Baseline differences were not statistically tested in accordance with the CONSORT guidelines. The LMM with random intercept and random slope was used to assess whether the changes of quantitative primary or secondary outcomes over time between propranolol and placebo group were significantly different. The generalized linear mixed model (GLMM) with random intercept was used to assess whether the changes of dichotomized outcomes over time between propranolol and placebo group were statistically significantly different. A two (measurements: baseline and follow-up) by two (conditions: intervention and placebo) Analysis of Variance (ANOVA) with repeated factor was used to compare the propranolol and placebo condition on physiological parameters (manipulation check) between baseline and follow-up. The significance level (alpha) was set at 5 and 95% confidence intervals are reported. Cohen's *d* was reported for each continuous outcome and the odds ratio was reported for dichotomous outcomes. A standardized formula to calculate Cohen's *d* based on LMM analyses is lacking. Therefore, for continuous outcomes, Cohen's *d* was calculated based on repeated measures Analysis of Variance (ANOVA) F-values regarding the group by time interaction with the formula $d\;=\;\sqrt{\left(F\left(\frac{n_{t}+n_{c}}{n_{t}n_{c}}\right)\left(\frac{n_{t}+n_{c}}{n_{t}+n_{c}-2}\right)\right)}$ (28). For within-group differences over time, Cohen's *d* was calculated based with the formula ***d*** **=** $\frac{{\bar{x}}_{t}-{\bar{x}}_{c}}{\sqrt{\frac{\left(n_{t}-1\right)s_{t}^{2}+\left(n_{c}-1\right)s_{c}^{2}}{n_{t}+n_{c}}}}$ (28). The effect size of GLMM is represented by the odds ratio of the interaction term between groups and time, which is transformed from log odds. A reliable chance index (RCI) for each individual participant was reported for the primary outcome measure, dental anxiety.

## Results

Between November 1, 2014, and December 31, 2018, 1,316 potential participants were screened for eligibility. Of these, 52 (4%) gave informed consent to participate and underwent randomized assignment (shown in Figure 1), 27 to receive propranolol and 23 to receive placebo. The inclusion was concluded after the week in which the minimum sample size of participants completed all phases of the study.

### Sociodemographic and Clinical Characteristics

The two study groups did not differ on any of the variables measured at baseline (see Table 1). In total, 36 participants (69% of the total group) took trial medication per protocol and completed the 1-month follow-up (shown in Figure 1).

**Table 1:** Baseline demographic and clinical characteristics of patients assigned to the propranolol and placebo groups.

		**Propranolol group (*****n*** **=** **19)**		**Placebo group (*****n*** **=** **17)**	
Number of participants		19		17	
		**Mean**	**SD**	**Mean**	**SD**
Age		29.26	10.84	26.71	8.46
Time since index trauma (years)		11.68	8.25	6.75	6.30
Dental anxiety (S-DAI) total score at first dental extraction		32.11	7.26	31.59	7.53
		**Proportion**		**Proportion**	
Index trauma dentistry related		12/19	63.2%	12/17	70.6%
Female gender		16/19	84.2%	13/17	76.5%
Finds extraction extremely anxiety provoking		9/19	47.4%	6/17	35.3%
Level of education	High school (4y)	4/19	21.0%	2/17	11.8%
	High school (5y)	5/19	26.3%	7/17	41.2%
	High school (6y)	4/19	21.0%	2/17	11.8%
	College	1/19	5.3%	3/17	17.6%
	University	5/19	26.3%	3/17	17.6%
Relationship status	Single	9/19	47.4%	10/17	58.8%
	Divorced	1/19	5.3%	0/17	0.0%
	Widow (er)	0/19	0.0%	1/17	5.9%
	Living apart	2/19	10.5%	2/17	11.8%
	Living together	6/19	31.6%	1/17	5.9%
	Married	1/19	5.3%	3/17	17.6%
Country of birth	The Netherlands	17/19	89.5%	15/17	88.2%
	Caribbean	0/19	0.0%	1/17	5.9%
	Morocco	1/19	5.3%	0/17	0.0%
	Asian	0/19	0.0%	1/17	5.9%
	European (other)	1/19	5.3%	0/17	0.0%
Specific phobia diagnosis at screening (Phobia Checklist)		5/19	26.3%	5/17	29.4%
Specific phobia diagnosis at first dental extraction (DSM-IV TR)		4/19	21.0%	6/17	35.3%
Specific phobia diagnosis at first dental extraction (DSM 5)		8/19	42.1%	9/17	52.9%

### Manipulation Check, Test of Blinding and Reactivation of the Fear Memory

#### Manipulation Check

**Figure 2:**
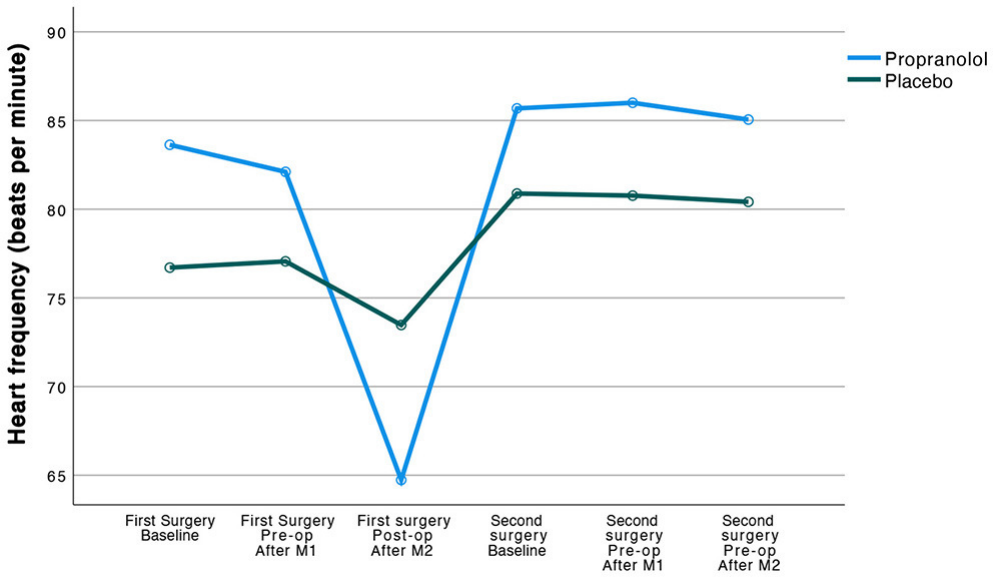
Observed trajectories of the heart frequency (beats per minute) as a function of treatment group.

Patients responded physiologically to the propranolol that was administered to them in that the propranolol group compared to their counterparts in the placebo group showed a significantly higher reduction in heart frequency (HF; −20 vs. −3 respectively, *F*_(1, 47)_ = 2,235.35, 95% CI [2,018.00, 452.71], *p* < 0.001), and in mean arterial pressure (MAP; −2 vs. +5, *F*_(1, 47)_ = 3,627.38, 95% CI [3,285.22, 3,969.54], *p* < 0.001), over time (from before to 1 h after ingestion of study medication). Observed trajectories of the HF as a function of treatment groups are displayed in Figure 2.

#### Test of Blinding

Among those in the propranolol group 15/26 (58%) suspected to have received propranolol vs. 8/23 (35%) among those in the placebo group; which was not statistically significant (difference = 22.91, 95% CI [−4.28, 50.10], χ^2^ (1) = 2.572, *p* = 0.11).

#### Reactivation of Unintentional Fear Memory by Anticipating the Dental Extraction

In the week prior to the first dental extraction, the mean IES-R score was 30.14.

### Dental Anxiety and Intra-operative State Anxiety

**Figure 3:**
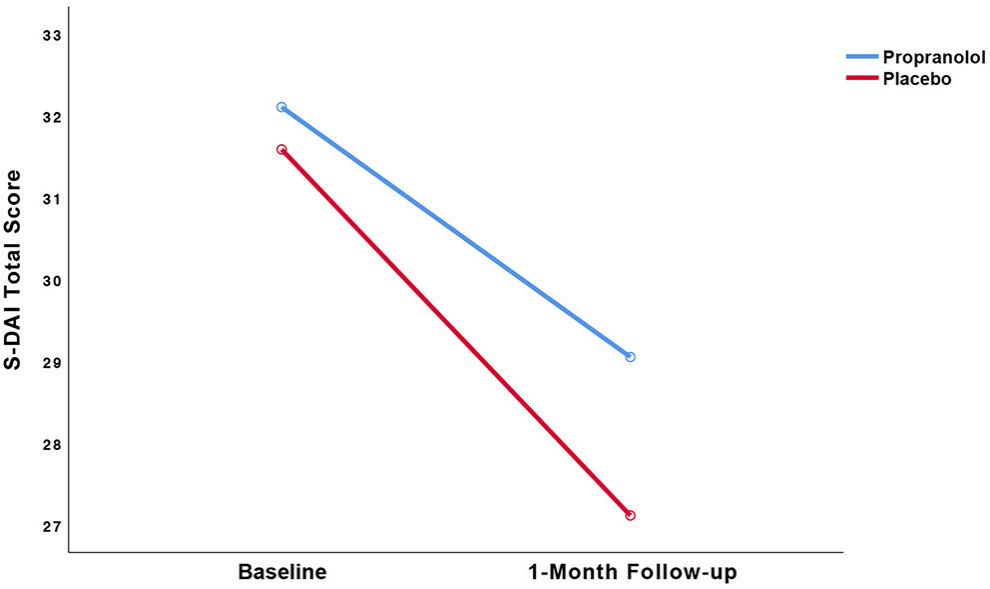
Observed trajectories of the dental anxiety (S-DAI) scores as a function of treatment group.

**Figure 4:**
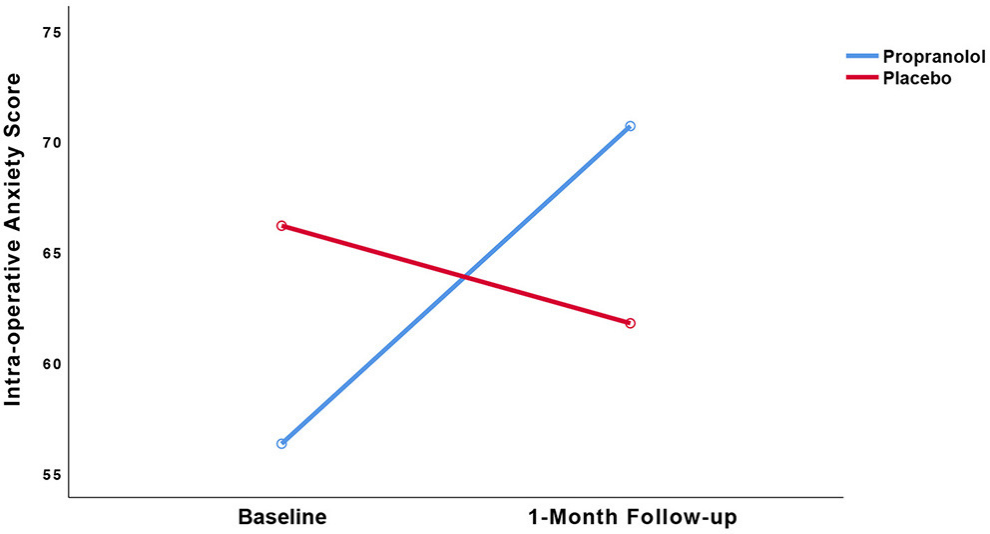
Observed trajectories of the intra-operative state anxiety score as a function of treatment group.

Results of the LMM analysis of the primary (i.e., dental anxiety) and secondary (i.e., intra-operative state anxiety) outcomes with the statistics for the group by time interaction are displayed in Table 2 and Figures 3, 4. Table 2 presents the means and standard deviations, LMM test statistics, effect sizes (d) for group by time interaction for these two outcome measures, and the *p*-value for the group by time interaction, analyzed by the linear mixed model analysis.

**Table 2:** Scores (Mean and SD) at baseline and 1-month follow-up, group by time interaction and between-group effect size.

**Measures**		**Propranolol group (reference group)**				**Placebo group**				**Group by time interaction**				**Effect sizes of propranolol** **vs. placebo group**
														**At 1-month follow-up**
		**Baseline score**	**SD**	**Follow-up score**	**SD**	**Baseline score**	**SD**	**Follow-up score**	**SD**	**Coefficient**	***95%-CI***		***p***	
											**Lower bound**	**Upper bound**		
S-DAI	*n*	19		19		17		17						
		32.11	7.26	29.05	8.82	31.59	7.53	27.12	6.55	−1.42	−5.54	2.70	0.50	0.23
Intra-operative state anxiety score	*n*	19		19		17		17						
		56.32	21.55	70.68	27.18	66.18	27.91	61.76	26.99	−18.78	−40.20	2.64	0.09	0.59
Specific phobia diagnoses	*n*	19		19		17		17						
		26.30%		21.05%		29.41%		5.88%		−2.50	−6,71	1.72	0.25	0.082

#### Dental Anxiety

The pre- to post-treatment effect sizes, respectively, were 0.39 in the propranolol group and 0.65 in the placebo group. LMM showed no statistically significant interaction between group and time (*p* = 0.50). Paired *t*-tests detected statistically significant within-group reduction in S-DAI scores in both the propranolol group (11% decrease, *t*_(19)_ = 2.135, 95% CI [0.05, 6.06], *p* < 0.05, *d* = 0.39) and in the placebo group (16% decrease, *t*_(17)_ = 2.914, 95% CI [1.22, 7.72], *p* = 0.01, *d* = 0.65). The RCI for each individual participant is displayed in Table 3. The RCI was significant in 9/19 participants in the propranolol group and in 10/16 participants in the placebo group.

**Table 3:** Reliable change index for primary outcome measure (S-DAI score).

**Participant number**	**Randomization allocation**	**x1**	**x2**	**x2 – x1**	**s_**x**_**	**r_**t**_**	**SE_**M**_**	**s_**t**_**	**SE_**diff**_**	**RCI**	**Sig**.
708	propranolol	24	33	9	7.535	0.88	2.610	6.2	3.037	2.9631	Sig.
786	propranolol	27	27	0	7.535	0.88	2.610	6.2	3.037	0.0000	N/S
860	propranolol	35	28	−7	7.535	0.88	2.610	6.2	3.037	−2.3046	Sig.
942	propranolol	30	23	−7	7.535	0.88	2.610	6.2	3.037	−2.3046	Sig.
945	propranolol	41	43	2	7.535	0.88	2.610	6.2	3.037	0.6585	N/S
947	propranolol	34	28	−6	7.535	0.88	2.610	6.2	3.037	−1.9754	Sig.
949	propranolol	28	28	0	7.535	0.88	2.610	6.2	3.037	0.0000	N/S
950	propranolol	29	15	−14	7.535	0.88	2.610	6.2	3.037	−4.6093	Sig.
953	propranolol	33	20	−13	7.535	0.88	2.610	6.2	3.037	−4.2800	Sig.
954	propranolol	38	39	1	7.535	0.88	2.610	6.2	3.037	0.3292	N/S
957	propranolol	33	20	−13	7.535	0.88	2.610	6.2	3.037	−4,2800	Sig.
959	propranolol	45	45	0	7.535	0.88	2.610	6.2	3.037	0.0000	N/S
961	propranolol	40	38	−2	7.535	0.88	2.610	6.2	3.037	−0.6585	N/S
965	propranolol	41	41	0	7.535	0.88	2.610	6.2	3.037	0.0000	N/S
970	propranolol	16	18	2	7.535	0.88	2.610	6.2	3.037	0.6585	N/S
972	propranolol	22	27	5	7.535	0.88	2.610	6.2	3.037	1.6462	N/S
975	propranolol	33	26	−7	7.535	0.88	2.610	6.2	3.037	−2.3046	Sig.
979	propranolol	34	32	−2	7.535	0.88	2.610	6.2	3.037	−0.6585	N/S
982	propranolol	27	21	−6	7.535	0.88	2.610	6.2	3.037	−1.9754	Sig.
702	placebo	39	27	−12	7.535	0.88	2.610	6.2	3.037	−3.9508	Sig.
709	placebo	32	23	−9	7.535	0.88	2.610	6.2	3.037	−2.9631	Sig.
859	placebo	42	27	−15	7.535	0.88	2.610	6.2	3.037	−4.9385	Sig.
943	placebo	27	35	8	7.535	0.88	2.610	6.2	3.037	2.6339	Sig.
944	placebo	43	43	0	7.535	0.88	2.610	6.2	3.037	0.0000	N/S
948	placebo	20	26	6	7.535	0.88	2.610	6.2	3.037	1.9754	Sig.
952	placebo	26	17	−9	7.535	0.88	2.610	6.2	3.037	−2.9631	Sig.
955	placebo	29	26	−3	7.535	0.88	2.610	6.2	3.037	−0.9877	N/S
956	placebo	30	30	0	7.535	0.88	2.610	6.2	3.037	0.0000	N/S
958	placebo	22	19	−3	7.535	0.88	2.610	6.2	3.037	−0.9877	N/S
960	placebo	31	25	−6	7.535	0.88	2.610	6.2	3.037	−1.9754	Sig.
964	placebo	38	36	−2	7.535	0.88	2.610	6.2	3.037	−0.6585	N/S
966	placebo	35	29	−6	7.535	0.88	2.610	6.2	3.037	−1.9754	Sig.
968	placebo	19	18	−1	7.535	0.88	2.610	6.2	3.037	−0.3292	N/S
973	placebo	28	25	−3	7.535	0.88	2.610	6.2	3.037	−0.9877	N/S
976	placebo	41	27	−14	7.535	0.88	2.610	6.2	3.037	−4.6093	Sig.
980	placebo	35	28	−7	7.535	0.88	2.610	6.2	3.037	−2.3046	Sig.

*N/S, Not statistically significant (-1.96 > RCI < 1.96); RCI, Reliable Change Index = (x1 – x2) / S_diff_; r_t_, Reliability of the test (Cronbach's α); S-DAI, Short version of the Dental Anxiety Inventory with total test scores ranging from 9 to 45 (Aartman, 1998); SE_diff_, Standard Error of measurement of the difference = √ (2 · (SE_M_)^2^); SE_M_, Standard Error of the Mean = S_X_ · √ (1 –r_t_); Sig., Statistically significant (R > 1.96 or RCI < −1.96); s_t_, Standard deviation of the test; s_X_, Standard deviation of test takers' scores = (s_x2_ + s_x1_) / 2; x1, Baseline score; x2, Follow-up score; x2 – x1, Observed difference in score*.

#### Intra-operative State Anxiety

LMM showed no statistically significant interaction between group and time *(p* = 0.09). Regarding intra-operative state anxiety, no within-group differences were found in the propranolol group (25% increase, *t* = (19) = −1.809, 95% CI [−31.05, 2.31], *p* = 0.09, *d* = 0.60) or placebo group (7% decrease, *t*_(17)_ = 0.597, 95% CI [−11.26, 20.08], *p* = 0.56, *d* = 0.17).

### Diagnostic Status

No significant change in specific (i.e., dental) phobia diagnoses over time between the propranolol group (*p* = 1.00) and the placebo group could be detected (*p* = 0.13; Table 2).

### Other Dependent Variables

No adverse events related to the study medication were reported. Also, no statistically significant associations were found between the study groups and any of the other dependent variables, i.e., proportion of surgical removals during the first and second extraction session, proportion of wisdom tooth removals during the first and second extraction session, extended duration (>15 mins) of the first surgery, extended duration of the second surgery, difficulty of the first surgery, difficulty of the second surgery, nor complications related to the first surgery.

## Discussion

This is the first randomized controlled trial aimed to determine long-term effectiveness of propranolol administered peri-operatively during exposure to the actually feared situation in reducing symptom severity among patients with traumatically induced fears and phobias. The results showed no statistically significant difference on dental trait anxiety and intra-operative state anxiety reduction between the propranolol and placebo groups over time.

The present results are in accordance with preliminary PTSD studies showing that the traumatic script reactivation procedure is effective in reducing symptom severity scores in both propranolol and placebo groups (12, 29). Furthermore, the results are in apparent contrast with a randomized trial by Liu et al. (23) with 23 dentally phobic individuals, who found a significant but one-tailed effect of propranolol over placebo on intra-operative state anxiety reduction during dental treatment. However, Liu et al.'s primary outcome would not have reached statistical significance if analyzed two-tailed. In the same vein, the present study failed to find a significant effect of propranolol over placebo on trait anxiety symptom severity. To the best of our knowledge, until now, only three well-designed previous RCTs have evaluated propranolol's effects on fear memory reconsolidation blockade among patients with anxiety or trauma-related disorders (12, 30, 31), which have shown mixed results. Only one trial, by Brunet et al. [discussed in more detail in the introduction (12)], found a positive effect of propranolol in a clinically relevant sample of individuals diagnosed with PTSD. Conversely, two recent trials failed to replicate this effect among individuals classified as fulfilling the diagnostic criteria of a mental health condition. Roullet et al. (31) who replicated the traumatic memory reactivation from the initial trial by Brunet et al. (12) in a double-blind placebo-controlled trial among 66 adults with PTSD and comorbid major depression, found no differences between propranolol and placebo on PTSD symptom severity (31). The authors reported lower participant attrition (12%; 58 and 51 participants completed the 7-week and 3-month follow-up, respectively) (31) than Brunet et al. (12) 50%; dropout (30 participants completed 6-week follow-up). Another recent study that failed to replicate the effectiveness of propranolol in a relevant clinical sample was carried out by Elsey and Kindt (30) who performed a double-blind placebo-controlled trial among 36 individuals with arachnophobia, also using a reactivation procedure. They found a tendency for better outcomes in the placebo condition in that these individuals improved greater than the propranolol group on phobic behavior scores (30). Notably, most compelling evidence for propranolol's effects on human fear memory reconsolidation originates from laboratory trials by the same research group (3–8). Nonetheless, these authors stated that “recent findings have even called into question the replicability of the basic laboratory phenomenon” [p. 17 (30)]. The current study's findings in a sample with individuals suffering from a severe fear are entirely in line with these results, casting further doubts on earlier conclusions and the hopeful message that the single use of a pill could potentially block reconsolidation of clinically relevant fear memories by transforming disturbing memories into a neutral memory.

How then can the present findings be explained? The lack of statistical differences with a trend toward better results in the placebo group in the present study may be explained in hindsight by two possible mechanisms, i.e., (a) the absence of a prediction error and (b) the duration of memory reactivation. First, regarding the absence of a prediction error, a laboratory study by Sevenster et al. (5) showed that a mismatch between what is expected based on what is learned during initial conditioning vs. what occurs during retrieval, is a boundary condition for memory labilisation as it permits human fear memory to be reconsolidated with a new emotional valence. To this end, our data did show that the feared invasive dental treatment was experienced as highly aversive, and intra-operative state anxiety levels in the propranolol and the placebo groups were equally high, suggesting that no prediction error (i.e., absence of the expected feared situation) occurred. Lack of prediction error is further substantiated by a subgroup analysis for which we excluded the outliers (*n* = 4) who reported low levels of intra-operative anxiety during the first surgery. To our surprise, the placebo group showed a significantly greater decrease in intra-operative state anxiety scores over time than the propranolol group with a large effect size (coefficient = −19.90, 95% CI [−38.62, −1.18], *p* = 0.04; Cohen's *d* = 0.84), whereas in the propranolol group scores increased. This suggests that propranolol in the present trial may have actually inhibited extinction rather than disrupting reconsolidation (6) in absence of a prediction error. Therefore, in future studies it is needed to verify the occurrence of a prediction error, e.g., by means of self-report by participants. Second, regarding the duration of memory reactivation, a study by Bos et al. showed that if multiple prediction errors occur during a long reactivation session, extinction rather than reconsolidation is likely to be triggered as the dominant memory process (6). Reactivation sessions in laboratory studies are usually very short, but strong fear memory networks in anxiety disorders may require longer sessions (22). If the duration of fear memory reactivation was too long in our trial, and the trials by Roullet et al. (31) and Elsey and Kindt (30), it is hard to fathom why effectiveness could be shown of the 10–20 min imaginal trauma reactivation in the PTSD trial by Brunet et al. (12). In our study, the initial imaginal reactivation of 30 secs was followed by an ~15-mins surgery an hour later. Based on the results reported by Brunet et al. (12), the absence of a prediction error in our study, rather than prolonged duration of exposure, may likely account for the observed results.

### Strengths and Limitations

Importantly, this study's sample size was low, which increased the likelihood of false negative results (i.e., non-rejection of a false null hypothesis) when the true effect size is small—although trends were found for better outcomes in the placebo group. An important strength of our study is the high dosage of propranolol we applied as we deemed required for individuals with high trait anxiety given that older memories may be less sensitive to disruption, e.g., Soeter and Kindt (22), Elsey and Kindt (30). A second strength of the study is the timing of drug plasma levels within the critical therapeutic window of β-adrenoreceptor involvement 2–3 h after retrieval (32), while not permitting impairment of memory retrieval during initial imaginal retrieval (12, 31, 33).

### Conclusions

The results of this trial suggest that fear memory reactivation and administration of perioperative propranolol may not reduce dental anxiety in patients with high levels of fear of dental extractions. The findings do not support the hypothesis that propranolol is capable of inhibiting traumatic memory reconsolidation in clinical practice. Moreover, the findings suggest that propranolol in our study may have actually inhibited traumatic memory extinction, rather than disrupting its reconsolidation, in absence of a prediction error. To date it remains uncertain how promising findings of propranolol's putative reconsolidation blocking effects in Pavlovian fear conditioning laboratory trials can be best translated into clinically meaningful interventions, as this study and other recent trials (30, 31) suggest that this may be substantially more difficult than previously expected (9, 30).

## Data Availability Statement

The raw data supporting the conclusions of this article will be made available by the authors, without undue reservation.

## Ethics Statement

The studies involving human participants were reviewed and approved by Institutional Review Board of the Academic Medical Center (MEC AMC) approved this trial on July 24, 2014 (application 2013_343). The patients/participants provided their written informed consent to participate in this study.

## Author Contributions

AJ conceived the idea for the trial design. AJ and SS constructed the study protocol. SS, AJ, AW, and NS constructed the statistical analysis plan and performed the power analysis. SS and DT acquired the data. SS, NS, AW, RW, DT, JL, and AJ have been involved in drafting the manuscript and revising it critically. All authors have made substantial contributions to the conception or design of the work, or the acquisition, analysis, or interpretation of data for the work, in drafting the work or revising it critically, and have read and approved the final manuscript.

## Conflict of Interest

The authors declare that the research was conducted in the absence of any commercial or financial relationships that could be construed as a potential conflict of interest.

## Publisher's Note

All claims expressed in this article are solely those of the authors and do not necessarily represent those of their affiliated organizations, or those of the publisher, the editors and the reviewers. Any product that may be evaluated in this article, or claim that may be made by its manufacturer, is not guaranteed or endorsed by the publisher.
